# Altered Low Frequency Oscillations of Cortical Vessels in Patients with Cerebrovascular Occlusive Disease – A NIRS Study

**DOI:** 10.3389/fneur.2013.00204

**Published:** 2013-12-16

**Authors:** Dorte Phillip, Helle K. Iversen, Henrik W. Schytz, Juliette Selb, David A. Boas, Messoud Ashina

**Affiliations:** ^1^Danish Headache Center, Glostrup Hospital, University of Copenhagen, Glostrup, Denmark; ^2^Stroke Unit, Department of Neurology, Glostrup Hospital, University of Copenhagen, Glostrup, Denmark; ^3^Photon Migration Imaging Laboratory, Athinoula A. Martinos Center for Biomedical Imaging, Harvard Medical School, Boston, MA, USA

**Keywords:** cerebral autoregulation, low frequency oscillations, hypoperfusion, stroke, carotid artery disease, Doppler, near infrared spectroscopy

## Abstract

Analysis of cerebral autoregulation by measuring spontaneous oscillations in the low frequency spectrum of cerebral cortical vessels might be a useful tool for assessing risk and investigating different treatment strategies in carotid artery disease and stroke. Near infrared spectroscopy (NIRS) is a non-invasive optical method to investigate regional changes in oxygenated (oxyHb) and deoxygenated hemoglobin (deoxyHb) in the outermost layers of the cerebral cortex. In the present study we examined oxyHb low frequency oscillations, believed to reflect cortical cerebral autoregulation, in 16 patients with both symptomatic carotid occlusive disease and cerebral hypoperfusion in comparison to healthy controls. Each hemisphere was examined with two NIRS channels using a 3 cm source detector distance. Arterial blood pressure (ABP) was measured via a finger plethysmograph. Using transfer function analysis ABP-oxyHb phase shift and gain as well as inter-hemispheric phase shift and amplitude ratio were assessed. We found that inter-hemispheric amplitude ratio was significantly altered in hypoperfusion patients compared to healthy controls (*P* = 0.010), because of relatively lower amplitude on the hypoperfusion side. The inter-hemispheric phase shift showed a trend (*P* = 0.061) toward increased phase shift in hypoperfusion patients compared to controls. We found no statistical difference between hemispheres in hypoperfusion patients for phase shift or gain values. There were no differences between the hypoperfusion side and controls for phase shift or gain values. These preliminary results suggest an impairment of autoregulation in hypoperfusion patients at the cortical level detected by NIRS.

## Introduction

Cerebral autoregulation is an intrinsic protective mechanism of the cerebral vessels to insure hemodynamic stability despite changes in the systemic blood pressure (1). A response of the middle cerebral artery velocity (Vmca) as assessed by transcranial Doppler (TCD) after spontaneous changes in arterial blood pressure (ABP) in the low frequency range is described as spontaneous cerebral autoregulation (2) and has become an established method of assessing cerebral autoregulation in cerebrovascular diseases (3, 4). While there is no gold standard for assessing spontaneous cerebral autoregulation it has been commonly examined by analysis of phase shift and gain (amplitude ratio) between ABP measured by the finger cuff method and 0.1 Hz low frequency oscillations (LFOs) of Vmca, measured by TCD (2). Examination with TCD allow for the major arteries to be investigated, but does not directly assess smaller vessels.

Instead near infrared spectroscopy (NIRS) may be used as a non-invasive optical method to investigate regional changes in oxygenated (oxyHb) and deoxygenated hemoglobin (deoxyHb) in the outermost layers of the cerebral cortex. Beyond its widespread use for task-related functional imaging, it is also emerging as a novel modality to measure spontaneous cerebral oscillations and derive information about dynamic cerebral autoregulation (2, 5–7). We have previously shown, that LFOs phase shift between ABP and NIRS-derived oxyHb showed little inter-hemispheric variability in healthy subjects (8). Thus, NIRS may be an ideal tool to measure subtle inter-hemispheric differences in spontaneous LFOs of oxyHb in cortical vessels in patients with hemodynamic impairment.

In the present study we examined cortical cerebral autoregulation by measuring oxyHb LFOs in patients with both symptomatic carotid occlusive disease and cerebral hypoperfusion. We compared hypoperfusion side versus the contralateral side and one side in healthy controls for phase shift and gain parameters. Furthermore, we compared hypoperfusion patients versus healthy controls for inter-hemispheric phase shift and amplitude ratio.

## Materials and Methods

### Subjects

We recruited 16 patients (14 male and 2 female, mean age 65, range 54–78) diagnosed with symptomatic carotid occlusion and hypoperfusion (Table 1). All patients were diagnosed with hypoperfusion based on SPECT with or without an acetazolamide challenge. The median time from the first symptoms occurred to examination was 18 months (range 1–108). For comparison, we used data from a group of 44 healthy controls (21 male, 23 female, mean age 47 years, range 28–69 years), which have previously been published (8).

**Table 1:** **Patient detail**.

Sub	Neurological examination	CT/MRI	Diagnosis	SPECT	Stenosis hypoperfusion side (%)	Stenosis contralateral side (%)
1	Normal	Normal	TIA	Unilat	80–99	60–79
2	Normal	Basal ganglia^a^	TIA	Unilat	80–99	0
3	Normal	Normal	TIA	Unilat	100	0
4	Hemiparesis	Frontal/occipital	Infarct	Unilat	100	0
5	Normal	Subcortical^a^	Infarct	Unilat	100	0
6	Normal	Normal	TIA	Unilat	100	0
7	Normal	Parietal/temporal	Infarct	Unilat	100	60–79
8	Normal	Parietal	Infarct	Unilat	100	0
9	Normal	Normal	TIA	Unilat	100	0
10	Hemiparesis	Frontal	Infarct	Global	0	0
11	Normal	Normal	TIA	Global	100	0
12	Normal	Normal	TIA	Unilat	0	0
13	Normal	Parietal/occipital	Infarct	Unilat	0	0
14	Normal	Parietal	Infarct	Unilat	0	0
15	Hemiparesis	Frontal	Infarct	Unilat	100	0
16	Normal	Normal	TIA	Unilat	100	0

*Unless otherwise stated, neurological deficits and imaging findings are on the hypoperfusion side. Diagnosis is based on clinical examination and imaging. CT/MRI, place of lesion; CT, computer tomography; MRI, magnetic resonance imaging; TIA, transitory ischemic attack on the hypoperfusion side; Infarct, infarct on the hypoperfusion side; SPECT, single photon emission computer tomography*.

*^a^ CT/MRI with signs of older infarction*.

The Biomedical Research Ethics committee in the Capital Region of Denmark approved the study (H-B-2008-088). All subjects gave informed consent to participate in the study, which was undertaken in accordance with the Helsinki Declaration of 1964, as revised in Edinburgh in 2000.

### Near infrared spectroscopy

Near infrared spectroscopy measurements were performed using continuous wave NIRS (NIRS2; TechEn Inc., Milford, MA, USA). The NIRS optodes were placed with two sources and four detectors on each side of the head with a distance of 3 cm between source and detector, each light source emitting two different wavelengths (690 and 830 nm). On each hemisphere, one source was placed on the forehead with one detector placed laterally, avoiding the midline sinus. The other source was placed on C3/C4 according to the International 10–20 system of EEG with the three detectors in front, above, and behind the source. Thus, the detectors measured the cerebral cortex in the territory supplied by the middle cerebral artery (MCA). NIRS recordings were acquired at a sampling rate of 200 Hz.

### Arterial blood pressure

Continuous non-invasive ABP recording was achieved via a finger plethysmograph (CNAP500) using the subjects left hand, positioned at heart level. Data from the finger plethysmograph was stored with the NIRS data for subsequent off-line analysis.

### Procedures

Before the experiments, each participant underwent a general physical and neurological examination. All experiments were performed with the patients placed in a supine position in a silent room with a constant temperature and the light dimmed. After 15 min of rest in the supine position, data acquisition was started with a 10 min trial of spontaneous breathing. This was followed by a 5 min trial with paced breathing at a rate of six respiration cycles per minute (0.1 Hz).

### Data analysis and statistics

Data are presented as mean ± SD. All signals were analyzed in successive 50% overlapping time segments of 100 s. The NIRS light intensity at 690 and 830 nm were first converted to time series of variations in oxyHb concentrations using the modified Beer–Lambert law. On each time segment, we then computed the power spectra of all signals (ABP, oxyHb), the coherence spectra for ABP-oxyHb and the complex transfer function for ABP-oxyHb (The Math Works, Inc., Natick, MA, USA) The phase shift and gain between ABP oscillations and oxyHb were obtained as the phase and absolute value of the complex transfer function respectively. The LFO frequency was selected as the frequency with maximal ABP oscillation power in the 0.09–0.11 Hz range. In this manner, for each run we obtained 5 (5 min run) or 11 (10 min run) overlapping segments each characterized by a coherence value, a phase shift, and a gain for ABP-oxyHb. As in our previous study we selected the segments for which the coherence was above an arbitrary threshold of 0.7 (8), and averaged the gain (linear average) and the phase shift (circular average) over these selected segments. If the total number of segments with coherence above 0.7 was less than 3 for a specific run, we discarded that run.

In the healthy controls the inter-hemispheric phase shift (LFO time difference between hemispheres) and amplitude ratio (LFO amplitude ratio between hemispheres) were obtained. In patients with hypoperfusion the inter-hemispheric phase shift was defined as the phase shift between the hypoperfusion and contralateral sides. Thus, a positive phase shift implied that the hypoperfusion side was ahead in time compared to the contralateral side. The inter-hemispheric amplitude ratio was defined as the amplitude ratio of contralateral/hypoperfusion sides. Thus, an amplitude ratio above 1 implied that the amplitude was lower on the hypoperfusion side compared to the contralateral side.

We also analyzed the absolute values of inter-hemispheric phase shift and inter-hemispheric amplitude ratio percentage difference, to examine the inter-hemisphere synchronicity and amplitude differences. These analyses investigate whether the inter-hemispheric phase synchronicity and the inter-hemispheric amplitude differences are significantly different in the healthy group and the hypoperfusion group, independently of whether the hypoperfusion side is behind or ahead in time or whether it presents larger or smaller amplitude of oscillations in comparison to the contralateral side.

Given that the hypoperfusion patients were clinically heterogeneous, we divided them into two groups: (1) Patients with unilateral hypoperfusion and ipsilateral occlusion or stenosis of internal carotid artery (*n* = 9) and (2) Patients with global hypoperfusion (*n* = 2) unilateral hypoperfusion without internal carotid artery occlusion (*n* = 3) or hypoperfusion treated with EC-IC bypass before the examination (*n* = 2).

The primary end-points were to compare for phase shift and gain for:

(A) Hypoperfusion side versus contralateral side in group 1 patients.
(B) Hypoperfusion side in group 1 patients versus healthy controls.
(C) Direct inter-hemispheric phase shift and amplitude ratio in group 1 patients versus healthy controls.

Near infrared spectroscopy values between hemispheres were compared for hypoperfusion patients using the non-parametric Wilcoxon signed-rank test. NIRS values between patients and healthy controls were compared using multiple regression analysis with age, gender, and hypoperfusion patient/healthy control status as independent parameters.

All analyses were performed with SPSS (18.0). We made no adjustment for multiple analyses. Thus, the level of significance at 0.05 was accepted for each comparison.

## Results

All patients completed the study, but one subject in group 1 was only examined with forehead recordings due to technical limitations.

In group 1 the percentage of recordings excluded because of low coherence (less than three windows) from the forehead were 22% on the hypoperfusion side (seven subjects remaining for analysis), and 44% (five subjects remaining for analysis) on the contralateral side which was not statistically different from healthy controls (24% excluded), *P* = 0.711. The percentage excluded from the parietal recordings was 63% on the hypoperfusion side and 29% on the contralateral side, which was not statistically different (*P* = 0.804). Because of the low number of patients and the low coherence in the parietal recordings, analysis was based on the results from the forehead. For the inter-hemispheric relation between hemispheres, 22% of the forehead recordings had low coherence in hypoperfusion patients (seven subjects remaining for analysis), and 7% in healthy controls, *P* = 0.200. With paced breathing at 0.1 Hz we attempted to modulate the LFOs, but it showed very low coherence in general, and was therefore not analyzed further.

### Hypoperfusion versus contralateral side

**Figure 1:**
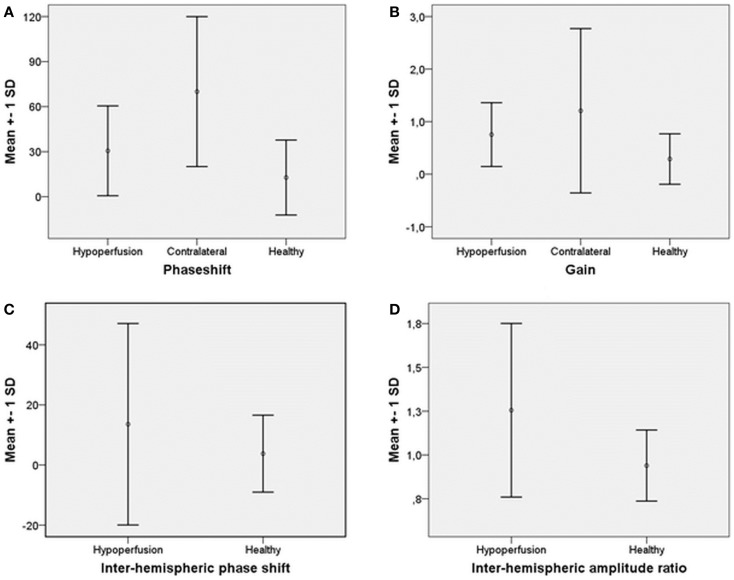
**Mean and ±SD of (A) phase shift ABP-oxyHb on hypoperfusion side, contralateral, and for healthy**. **(B)** Gain ABP-oxyHb on hypoperfusion side, contralateral, and for healthy. **(C)** Inter-hemispheric phase shift hypoperfusion to contralateral side and for healthy. **(D)** Inter-hemispheric amplitude ratio contralateral/hypoperfusion side and for healthy.

The LFO ABP-oxyHb phase shift on the hypoperfusion side (31 ± 30°), tended to be higher than on the contralateral side (70 ± 50°), *P* = 0.080, Figure 1A. There was no significant difference in ABP-oxyHb gain between the hypoperfusion side (0.7 ± 75%), and the contralateral side (1.2 ± 133%), *P* = 0.225, Figure 1B.

### Hypoperfusion patients versus healthy controls

Forehead measurements from the hypoperfusion side were compared to the left side of healthy controls. We found no difference in phase shift between patients (31 ± 30°) and healthy controls (13 ± 25°), *P* = 0.186, Figure 1A. There was also no difference in gain between patients (0.7 ± 75%) and healthy controls (0.3 ± 190%), *P* = 0.389, Figure 1B.

### Inter-hemispheric relation in patients versus healthy controls

Mean phase shift from the hypoperfusion side to the contralateral side (14 ± 34°) was not different compared to left to right phase shift in healthy controls (4 ± 13°), *P* = 0.463, Figure 1C. There was a tendency toward higher inter-hemispheric absolute phase shift in hypoperfusion patients (23 ± 27°) compared to healthy controls (9 ± 10°), *P* = 0.061.

There was a significantly higher mean amplitude ratio for contralateral/hypoperfusion side in patients (1.3 ± 0.5) compared to healthy controls (0.9 ± 0.2), *P* = 0.010, Figure 1D. The mean percentage amplitude difference in hypoperfusion patients (44 ± 32%) was also higher compared to healthy controls (16 ± 14%), *P* = 0.003.

Phase shift and gain values for group two patients are presented in Table 2.

**Table 2:** **Phase shift and gain values for the patients with global hypoperfusion (sub 10 and 11), the patients without stenosis and occlusion (sub 12–14), patients that were treated with EC-IC bypass before examination (sub 15–16) and prediction intervals for healthy controls**.

Subject	Phase shift			Gain		
	Hypoperfusion	Contralateral	Direct	Hypoperfusion	Contralateral	Direct
10	LC	LC	12	LC	LC	1.1
11	47	53^a^	−8	0.6	1.0	1.3
12	10	13	−9	2.0	1.9	1.1
13	88	53	−3	1.4	0.9	1.3
14	LC	LC	11	LC	LC	0.8
15	LC	14	33	LC	8.9	1.1
16	−20	29	−48	0.7	0.6	1.2
HC PI		−45, 75	−21, 29		0.03, 2.2	0.6, 1.5

*HC, healthy controls; LC, low coherence (less than three windows); PI, prediction interval*.

*^a^ Hemisphere with hypoperfusion, but no stenosis or occlusion*.

## Discussion

We found that inter-hemispheric amplitude ratio was significantly altered in hypoperfusion patients compared to healthy controls, due to relatively lower amplitude on the hypoperfusion side. There was a trend toward increased phase shift in inter-hemispheric phase shift in patients. We found no statistical difference between hemispheres for hypoperfusion patients for phase shift or gain, and no difference for hypoperfusion side compared to healthy controls for phase shift or gain values.

Assessing cerebral autoregulation through examination of spontaneous LFO with NIRS or TCD has the advantage of being without discomfort during recording, which ensure high compliance from the patients. Furthermore, NIRS directly investigates local changes in the cortical vessels, opposed to measurements of changes in Vmca via TCD.

Previous studies of LFOs in carotid artery stenosis and occlusion has primarily investigated phase shift and gain between Vmca and ABP during spontaneous (3, 9) or paced breathing (10–12). These studies have showed a significantly lower phase shift for both spontaneous (9, 13) and paced breathing (12) on the affected side. Interestingly, LFOs gain between MAP and Vmca has also been shown to decrease with increasing levels of stenosis (13). Phase shift was examined in combination with CO_2_ reactivity by Reinhard et al. and both were found to be reduced on the affected side, but no correlation was found between the two measurements (10). LFOs phase shift has also shown equally good sensitivity and specificity for detecting high-grade carotid stenosis and occlusion as conventional tests such as CO_2_ vasoreactivity (13) and tilt test (3), making it at promising tool for CA assessment. Cortical vessels appear to be affected by large artery stenosis. Thus, Ziyeh et al. demonstrated decreased blood oxygen level dependent (BOLD) effect within the internal carotid artery territory in patients with high-grade carotid stenosis and occlusion, which also had impaired CO_2_ reactivity measured with TCD (14). Only one study has examined changes in LFO phase shift with NIRS and TCD simultaneously in asymptomatic patients using paced breathing (6). The study showed that both ABP-oxyHb and ABP-TCD phase shift was right-shifted in comparison to the contralateral side. Thus, the study suggests that oxyHb measurements may detect similar changes suggestive of cerebral autoregulation impairment as Vmca measurements (6).

In the healthy brain, the inter-hemispheric phase shift is expected to be close to 0° and the inter-hemispheric amplitude ratio close to 1 (8), as a sign of the two hemispheres being synchronous and at the same level of cerebral autoregulation. In the present study, the direct inter-hemispheric relation showed a higher amplitude ratio and percentage amplitude difference in patients with hypoperfusion and ipsilateral carotid artery stenosis or occlusion compared to healthy controls. This novel finding is likely due to arterioles being compensatively dilated in the hypoperfusion hemisphere by the demand of adequate perfusion and oxygen delivery distal to the stenosis. This could affect the capacity for LFO and thereby decrease the amplitude on the ipsilateral hemisphere distal to the stenosis. This mechanism is supported by previously mentioned studies showing reduced CO_2_ vasoreactivity on the side of carotid stenosis or occlusion (9, 10). Furthermore, a previous NIRS study (15) reported that frontal OxyHb LFOs were reduced in patients with previous ischemic infarction compared to healthy controls. This suggests that cerebrovascular disease may result in decreased vasodilatory capability or increased vascular stiffness.

The absolute inter-hemispheric phase shift showed a trend toward less synchronicity between hemispheres in hypoperfusion patients compared to healthy controls. However, there was no significant difference when analyzing the inter-hemispheric phase shift from hypoperfusion to contralateral side. Thus, there was no indication of oscillations on the hypoperfusion side being systematically ahead or behind in time to oscillations on the healthy side. However, these results need to be interpreted with care due to the low number of subjects, which could lead to type 2 errors.

The strength of analyzing inter-hemispheric differences is that each subject serves as its own reference. ABP-oxyHb values will be more sensitive to errors due to the actual geometry of each head. Thus, by measuring inter-hemispheric differences, we are less sensitive to variability between subjects and thereby minimize model errors.

Group 2 was a very heterogenous group and statistic comparison within the group was not possible. When looking at the predictive intervals obtained from healthy subjects, the values found in group 2 were mostly within normal range, which would be expected for ABP-oxyHb gain because of the very wide normal range, but not for phase shift and inter-hemispheric values. The long time period from debut of symptoms to examination, the EC-IC bypass for two of the subjects and thereby possible normalization of LFOs could be part of the explanation of these normal values. Involvement of both hemispheres results in normal inter-hemispheric values.

The study has limitations which need to be acknowledged. The number of subjects in this study is low and it is therefore not possible to make any conclusions regarding the non-significant findings. The significant results in this study, was found in inter-hemispheric data compared to healthy controls, which requires access to normative data or evaluation over time. There are also potential confounding factors such as medication, recent stroke, or the level of collateral circulation that may affect CA assessed via LFOs analysis. In support of this, Reinhard et al. (16) reported decreased paced LFOs phase shifts with the recruitment of secondary collateral pathways, that is, a retrograde flow via the external carotid and ophthalmic artery and leptomeningeal collateral flow via the posterior cerebral artery. The significant inter-hemispheric changes found could potentially be due to movement artifacts resulting in decreased synchronicity between hemispheres as well as larger amplitude difference, but we have no indication of hypoperfusion patients having moved more during recording sessions. Furthermore, because of multiple comparisons, there is an increased risk of type 1 error. Coherence was very low in the present study, which resulted in exclusion of all paced breathing data. Our coherence limit is higher than in other groups (6). At present, there is no recognized standard for analyzing NIRS LFOs, and therefore it is unknown whether the lack of coherence during paced breathing is due pathophysiology, patient compliance, or technical issues.

In conclusion, our preliminary results suggest that inter-hemispheric gain was significantly altered in hypoperfusion patients compared to healthy controls and that inter-hemispheric phase shift showed a trend toward higher absolute phase shift in hypoperfusion patients compared to controls. Investigation of cortical vascular changes in hypoperfusion patients is feasible using NIRS and future studies are warranted.

## Author Contributions

Dorte Phillip contributed to design of the study, acquisition of data, analysis of data, and writing the paper. Helle K. Iversen contributed to design of the study, interpretation of data, and writing the paper. Henrik W. Schytz contributed to design of the study, acquisition of data, analysis of data, and writing the paper. Juliette Selb contributed to analysis and interpretation of data and writing the paper. David A. Boas contributed to analysis and interpretation of data and writing the paper. Messoud Ashina was the study promoter, contributed to design of the study, interpretation of data, and writing the paper.

## Conflict of Interest Statement

The authors declare that the research was conducted in the absence of any commercial or financial relationships that could be construed as a potential conflict of interest.
